# Acute Pericarditis and Pericardial Effusion in a Hypertensive COVID-19 Patient

**DOI:** 10.7759/cureus.10705

**Published:** 2020-09-29

**Authors:** Syeda Ghadeer Zehra Naqvi, Uzma Naseeb, Kainat Fatima, Sumaira Riffat, Anjuman Gul Memon

**Affiliations:** 1 Internal Medicine, Sindh Medical College, Jinnah Sindh Medical University, Karachi, PAK; 2 Cardiovascular Medicine, Jinnah Post Graduate Medical Center, Karachi, PAK; 3 Cardiology, Sindh Medical College, Jinnah Sindh Medical University, Karachi, PAK; 4 Physiology, Sindh Medical College, Jinnah Sindh Medical University, Karachi, PAK; 5 Internal Medicine, College of Medicine, Qassim University, Buraidah, SAU

**Keywords:** cardiac, cardiovascular, covid-19, hypertensive, pericarditis, pericardial effusion

## Abstract

Coronavirus disease 2019 (COVID-19) is a contagious disease that has a potential of causing cardiovascular illness. Cardiac outcomes of COVID-19 mainly include acute coronary syndrome, heart failure, and left ventricular dysfunction. However, pericardial involvement is very rare. Here, we present a case of pericarditis and pericardial effusion in a known hypertensive COVID-19 patient. Our case was a diagnostic dilemma as the literature review mentioned that cardiovascular manifestations are mostly reported in symptomatic and critically ill patients of COVID-19. However, this patient has no viral respiratory illness, and is otherwise healthy.

## Introduction

Coronavirus disease 2019 (COVID-19) is a contagious disease that can cause mild-to-moderate respiratory illness, ranging from flu, cough, dyspnea, chest tightness to more severe conditions including metabolic acidosis, severe respiratory distress syndrome, shock, and multi-organ failure leading to death [1,2]. Different clinical and epidemiological studies have demonstrated that cardiovascular manifestations including acute coronary syndrome, heart failure, and left ventricular dysfunction were present in the COVID-19 infected patients [3].

## Case presentation

A 55-year-old male presented to the emergency department with acute chest pain for the past 24 hours. There was no episode of fatigue, chest pain, and dyspnea in the past. The patient had no recent history of documented fever, cough, and flu. Clinical history of the patient revealed that he was a case of hypertension over a period of 10 years. The patient was taking antihypertensive drugs irregularly. He had a positive family history of myocardial infarction and hypertension in first- and second-degree relatives. He was living in a home with two active home-isolated cases of COVID-19 in the immediate family, and the patient was a caregiver of the infected family members. Extensive history of the patient was taken to rule out other diseases. Apart from hypertension, he had no other comorbidities, and there was no history of any major cardiac event in the past. Past medical, surgical, drug, and addiction history was not significant.

**Figure 1:**
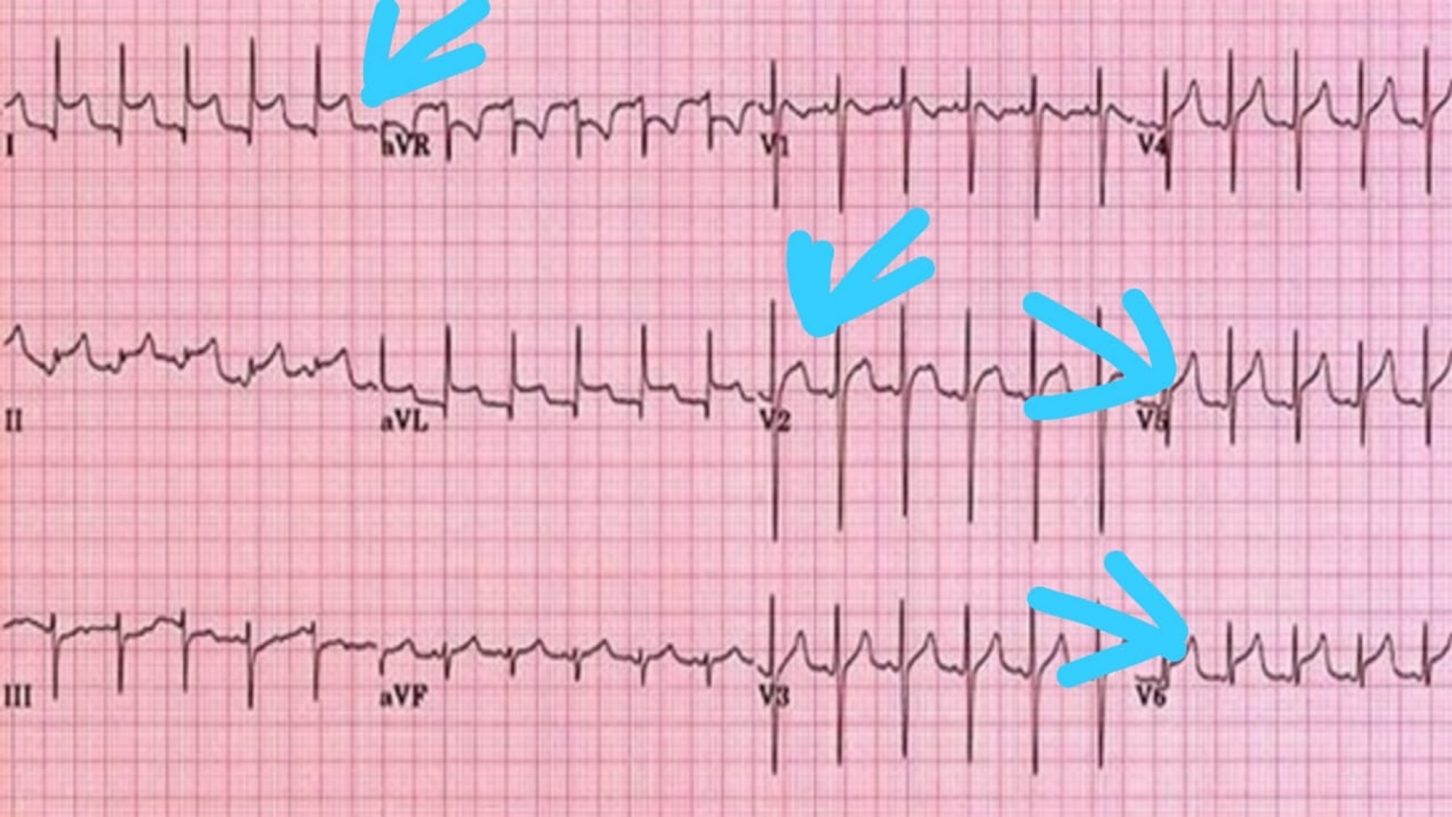
A 12-lead ECG showing widespread ST-segment elevation ECG, electrocardiogram

**Figure 2:**
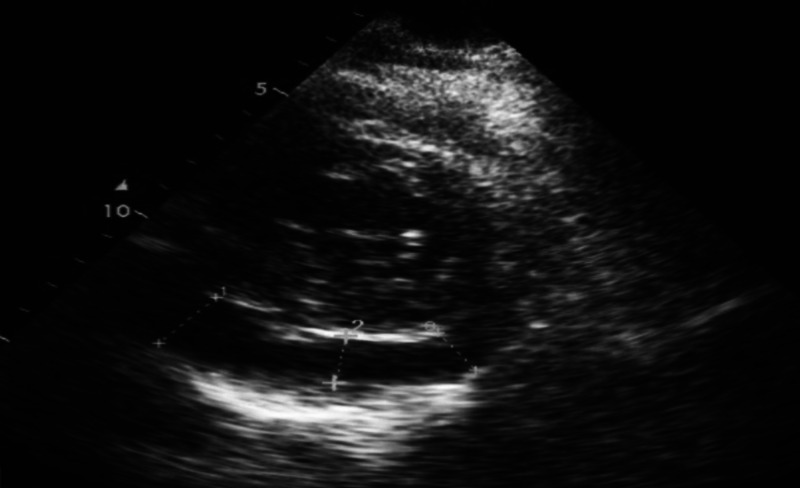
Echocardiography (parasternal view) demonstrating moderate pericardial effusion

On physical examination, his blood pressure was 85/55 mm Hg, pulse was 96 beats per minute, temperature was 98°F, and oxygen saturation was 98%. Chest auscultation finding was pericardial friction rub, a scratching sound best heard in systole. Electrocardiographic findings revealed ST-segment elevation in almost all of the leads (Figure 1). Echocardiographic examination showed mild-to-moderate pericardial effusion (Figure 2). Noticeable labs included leukocytosis and raised cardiac troponin I value of 0.05 ng/mL, which peaked to 90 ng/mL within the next few hours of hospital admission. On the basis of clinical history, nasopharyngeal swab test was performed, which resulted in a positive finding of severe acute respiratory syndrome virus (SARS-CoV) on polymerase chain reaction (PCR) assay. There were no respiratory symptoms throughout the clinical course. Clinical assessment on the basis of history and examination, positive family history of COVID-19, positive PCR assay, and specific findings on investigations supported the diagnosis of acute pericarditis secondary to COVID-19 illness. Indomethacin 25 mg three times daily and aspirin 600 mg six times daily were prescribed, and pericardiocentesis was planned. The patient had not received any treatment before coming to the emergency department.

## Discussion

Concerns of cardiac outcomes in COVID-19 patients have been highlighted relating to worst outcomes in severely symptomatic and critically ill patients of COVID-19, with the most frequent presentation of hypotension, sinus tachycardia, arteriovenous block, ventricular tachycardia, pericardial effusion, and heart failure [4]. However, pericardial involvement is rare with the therapeutic challenge. High-dose aspirin and nonsteroidal anti-inflammatory drugs (NSAIDs) are the mainstay of treatment [5].

Risk estimation of cardiovascular manifestations can be performed by evaluating cardiac risk factors and comorbidities of an infected patient [6]. Preexisting cardiovascular comorbidities are considered to be the major risk factor for the occurrence of cardiovascular events in COVID-19 patients with a higher proportion of hypertensive patients suffering from acute cardiac injury and heart failure [7].

Our case was a diagnostic dilemma as the literature review mentioned that cardiovascular manifestations are mostly reported in symptomatic and critically ill patients of COVID-19. However, our patient had no medical illness, past cardiac, or respiratory event, and was otherwise healthy. This study adds these valuable findings in the existing literature and sheds light on the cardiac manifestations in a silent carrier of COVID-19.

## Conclusions

This case highlighted cardiac outcomes in a hypertensive patient without any other comorbidities. Risk estimation of cardiovascular illness can be performed on the basis of comorbidities, cardiac risk factors, and family history of cardiac illness.

This case identifies the need of keeping high suspicion on caregivers of home-isolated COVID-19 infected patients with a positive history of hypertension and other comorbidities. Documentation and proper counseling of home-isolated patients and their caregivers will prevent this contagious illness. Furthermore, healthy elderly individuals living with active cases of COVID-19 should be highly encouraged to follow risk mitigation measures and be evaluated regularly for the risk of transmission of this contagious virus.
